# A scalable phenotyping approach for female floral organ development and senescence in the absence of pollination in wheat

**DOI:** 10.1242/dev.200889

**Published:** 2022-09-15

**Authors:** Marina Millan-Blanquez, Matthew Hartley, Nicholas Bird, Yann Manes, Cristobal Uauy, Scott A. Boden

**Affiliations:** ^1^ John Innes Centre, Norwich Research Park, Norwich NR4 7UH, UK; ^2^ European Molecular Biology Laboratory, European Bioinformatics Institute (EMBL-EBI), Wellcome Genome Campus, Hinxton, Cambridge CB10 1SD, UK; ^3^ KWS UK Ltd, Thriplow, Hertfordshire SG8 7RE, UK; ^4^ Syngenta France S.A.S., Le Jardin des Entreprises, 28 000 Chartres, France; ^5^ School of Agriculture, Food and Wine, Waite Research Institute, University of Adelaide, Glen Osmond 5064, South Australia, Australia

**Keywords:** Carpel development, Machine learning, Stigma, Ovary, Wheat, Hybrid breeding

## Abstract

In the absence of pollination, female reproductive organs senesce, leading to an irrevocable loss in the reproductive potential of the flower, which directly affects seed set. In self-pollinating crops like wheat (*Triticum aestivum*), the post-anthesis viability of unpollinated carpels has been overlooked, despite its importance for hybrid seed production systems. To advance our knowledge of carpel development in the absence of pollination, we created a high-throughput phenotyping approach to quantify stigma and ovary morphology. We demonstrate the suitability of the approach, which uses light-microscopy imaging and machine learning, for the analysis of floral organ traits in field-grown plants using fresh and fixed samples. We show that the unpollinated carpel undergoes a well-defined initial growth phase, followed by a peak phase in which stigma area reaches its maximum and the radial expansion of the ovary slows, and a final deterioration phase. These developmental dynamics were consistent across years and could be used to classify male-sterile cultivars. This phenotyping approach provides a new tool for examining carpel development, which we hope will advance research into female fertility of wheat.

## Teaser

**Summary:** A new automated phenotyping method for stigma and ovary development in wheat highlights the potential for genetic variation that could benefit hybrid breeding.

## INTRODUCTION

The fertilisation of the pistil by a pollen grain is a vital event in the life cycle of a flowering plant as it contributes to the reproductive fitness of a species. In grasses, the pistil (or carpel) typically consists of an ovary bearing two styles densely covered by a feathery and dry-type stigma (Walker, 1906; Heslop-Harrison and Shivanna, 1977). The stigmatic tissue plays a key role in successful fertilisation as it facilitates the interception and hydration of the pollen grain and mediates pollen tube growth into the stylodium branches towards the ovary containing the ovule (Heslop-Harrison, 1979; Edlund et al., 2004). After successful fertilisation, the ovary undergoes growth and differentiation to develop into a grain. Under favourable growing conditions, the duration of carpel receptivity (or functionality) does not present a serious limitation to seed formation in self-pollinating species, such as wheat (*Triticum aestivum*) or rice (*Oryza sativa*). However, environmental stresses such as heat and drought (Fábián et al., 2019; Onyemaobi et al., 2016; Mitchell and Petolino, 1988) or the absence of viable pollen [e.g. male sterile cultivars used in hybrid breeding (Kempe and Gils, 2011)] can affect normal seed set.

Female floral organs have developed a series of survival mechanisms to secure seed set in the absence of self-pollination by increasing the likelihood of receiving pollen from neighbouring male fertile plants. Indeed, this process (directly or indirectly) has been harnessed by breeders to produce hybrid seeds in crops like maize (*Zea mays*), rice, barley (*Hordeum vulgare*) and wheat. In maize, one of the survival strategies described to increase pollen capture is silk (i.e. stigma) emergence and elongation from the husk (Bassetti and Westgate, 1993), whereas in wheat, the radial expansion of the unfertilised ovary pushes the floret open, facilitating access to airborne pollen, a phenomenon known as the ‘second opening’ (Molnár-Láng et al., 1980; Okada et al., 2018). However, if pollination still does not occur after a specific duration of time, which varies between species (Primack, 1985; Ashman and Schoen, 1994), a series of developmental processes leads to the senescence of the floral organs and the irreversible loss of reproductive potential (Carbonell-Bejerano et al., 2010). For example, in several plants, the loss of papilla integrity has been regarded as one of the primary symptoms indicating the end of the floral receptive period and stigma senescence, which is often manifested by the shrunken appearance of the stigma (Gao et al., 2018; Okada et al., 2018; González et al., 1995). These senescence processes are coordinated by transcription factors, including *KIRA1* (*KIR1*) and *ORESARA1* (*ORE1*) in *Arabidopsis* (Gao et al., 2018). Similarly, the unfertilised ovary undergoes a series of morphological changes that converge in the lignification of the epidermal cells and eventual collapse of the ovary walls (Carbonell-Bejerano et al., 2010; Okada et al., 2018). In many of these studies, the phenotypic characterisation of these processes is time consuming and labour intensive, and is, therefore, usually performed only under controlled growing conditions and on a small number of plants. These phenotyping approaches, although extremely informative, are often not conducive for translation into breeding targets, for which the screening of large germplasm sets is required.

In recent years, high-throughput phenotyping technologies have provided new opportunities to phenotype a diverse range of plant species at various scales, ranging from cellular to tissue and organ levels (Pieruschka and Schurr, 2019; Furbank and Tester, 2011). For instance, machine-learning-based algorithms, like neural networks, have become an essential tool for reliably extracting morphological information and providing visual quantitative parameters of microscopy images. These approaches can be used in large-scale experiments, like those of crop breeding programmes, and provide a way to quantify the morphological changes of the developing carpel in the absence of pollination.

To advance our knowledge of carpel development in the absence of pollination, we developed a phenotyping approach for the quantification of stigma area and ovary diameter of field-grown plants by combining light microscopy and machine learning. We focused on bread wheat carpels due to the current need to improve outcrossing rates in hybrid breeding programmes (Selva et al., 2020) and the lack of knowledge on the dynamics of stigma and ovary development among male sterile (MS) wheat cultivars under production conditions in the field. We applied our phenotyping approach to three MS cultivars during two consecutive field seasons to gain insights into genetic and environmental variation for these two floral traits, and show that it is scalable to produce practical advances in breeding programmes.

## RESULTS

### Defining quantifiable parameters of late carpel development and senescence

**Fig. 1.**
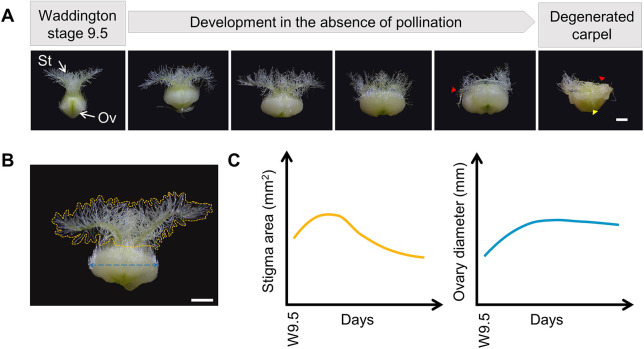
**Representative stages of late carpel development in the absence of pollination.** (A) Morphological changes observed in the stigma (St) and ovary (Ov) from Waddington stage 9.5 (approximate timing of ear emergence) until complete degeneration of the carpel (from left to right). Arrowheads indicate regions of the stigma (red) and ovary (yellow) where symptoms of cell degeneration are visible. (B) Diagram illustrating the morphological traits of interest. The yellow line delimits the area covered by the stigmatic hairs, the blue line depicts the diameter of the ovary. (C) Representative growth trends observed for the stigma hair area (yellow) and ovary diameter (blue) in the absence of pollination for field-grown plants. For the regression curves, we used a single exemplar MS cultivar (*n*=10-30 carpels sampled from six plants per timepoint, from a total of 8 timepoints). Scale bars: 1 mm.

To investigate the development of wheat carpels in the absence of pollination and determine the parameters that correlate with key phases of their life cycle, we used nuclear and cytoplasmic male sterile (NMS and CMS, respectively) plants. We imaged the unpollinated carpels of field-grown plants starting from Waddington stage 9.5 (W9.5, normally shortly after ear emergence; Fig. 1A), which corresponds with the most advanced developmental stage for an unpollinated carpel. W9.5 is shortly before the stage at which male fertile plants reach anthesis (W10) and release viable pollen on the receptive stigma surface. At W9.5, stigma branches are well elongated and spread outwards to generate the plumose architecture, whereas the unfertilised ovary shows a round shape (Fig. 1A). In subsequent timepoints after W9.5, the unfertilised ovary gradually expands horizontally leading to the second opening of the floret (as previously described by Okada et al., 2018 and Molnár-Láng et al., 1980). During this period of ovary growth, stigma branches continue to grow and quickly curve away from each other, increasing the stigma surface area and contributing to the extrusion of the unpollinated stigma outside the floret for the capture of airborne pollen. Towards the end of the time course, papilla cells of the stigma hairs start to lose turgor and become atrophied as the stigma degenerates (Fig. 1A, red arrowheads). Finally, the onset of stigma degeneration is followed by a slight and gradual decline in ovary radial size, causing the floret to close again (Molnár-Láng et al., 1980).

To quantify the observed morphological changes in these parameters, we imaged unpollinated carpels and manually delineated the area covered by the stigma hairs and the diameter of the ovary using Fiji (Fig. 1B). We distinguished a developmental pattern for stigma area characterised by an initial bell curve followed by a gradual reduction in area indicative of tissue deterioration. The ovary diameter gradually increased and reached a plateau with little changes in the diameter thereafter (Fig. 1C). More importantly, these patterns appear to be quite consistent across different cultivars and replicates (Fig. 5). Taken together, these findings suggest that stigma area and ovary diameter are promising parameters to quantitively track the life cycle of the unpollinated carpel.

### Overview of the approach

**Fig. 2.**
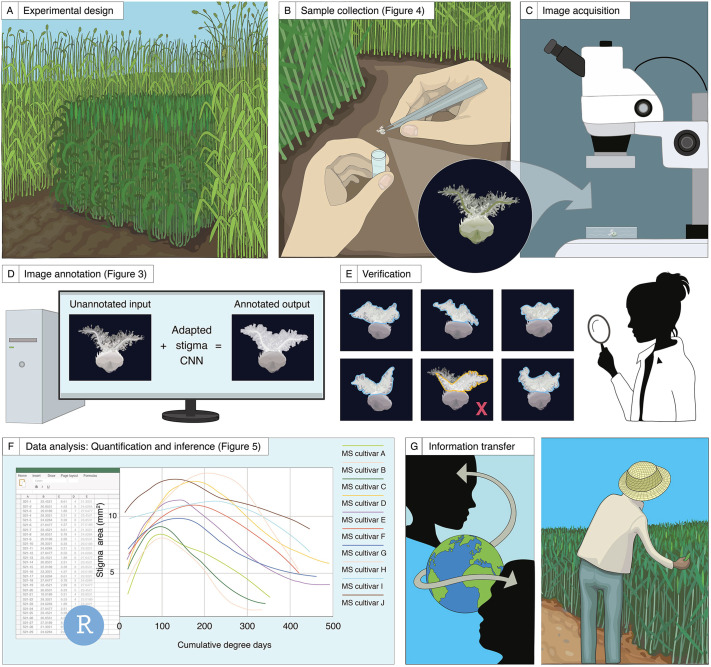
**Schematic representation of the proposed phenotyping approach for the study of carpel development in the absence of pollination under field conditions.** (A-C) Experimental design, sample collection and image acquisition. (D,E) Annotation of microscopy images and quantification of the stigma area (an example shown in D) and verification of the CNN outputs (E). (F,G) The applicability of the phenotyping approach to enhance our understanding of the post-anthesis behaviour of unpollinated carpels.

The rapid and accurate phenotyping of large numbers of field-grown plants represents a challenge for plant researchers. Here, to accelerate research into female floral traits, we propose a phenotyping approach that can be implemented in the screening of large populations, such as those of pre-breeding programmes (Fig. 2). This approach provides a visualisation and quantification toolbox for morphometric information of stigma area and ovary diameter. A summary of each step is provided below, and detailed descriptions can be found in the Materials and Methods.

#### Experimental design, sample collection and image acquisition

As our main aim was to study carpels in the absence of pollination, the first step is to prevent cross-pollination of MS plants in the field. To achieve this, different strategies can be used. For example, in this study, we grew sterile rye surrounding the experimental plots to create an effective pollen barrier from surrounding fertile plants (Fig. 2A; Fig. S1). When anthers from the outer florets of the central spikelets started to turn yellow and stigmatic branches started to spread outwards (W9.5), we tagged spikes to indicate the beginning of the time course. Depending on the location and scale of the experiment, logistical issues such as transport and preservation methods were also considered at the time of sampling. For sample collection, we carefully dissected wheat carpels from central spikelets in the field, which can be performed by eye as they are relatively large (Fig. 2B). Alternatively, we cut individual tillers between the uppermost and penultimate internode and transported them in water to the laboratory for carpel dissection. Once dissected, we stored the carpels in 95% ethanol and acetic acid (75% v/v) for image acquisition at a later timepoint, or fresh (non-fixed) specimens were imaged directly if tillers had been transported to the laboratory. We used a stereo microscope with an integrated camera to acquire the two-dimensional RGB image of the carpel against a black background (Fig. 2C). We used different magnifications and fields of view to capture the best representative plane of the carpel (Fig. S3). In the case of the fixed samples, we placed carpels in Petri dishes with 70% ethanol to preserve the feathery appearance of the stigma. Only one image per sampled carpel is required for subsequent steps.

#### Annotation of microscopy images and quantification of carpel traits

To process and perform quantitative analyses of the microscopy images, we trained a machine-learning (ML)-based algorithm to automatically and rapidly annotate and measure stigma area and ovary diameter (Fig. 2D). The trained networks return a set of annotated images alongside their shape descriptors, together with a CSV file containing the measurements of the analysed images in an output folder (see supplementary Materials and Methods for a step-by-step guide on how to use the trained networks). This step requires some manual curation wherein the user inspects, detects and corrects annotation errors, or removes corrupted images (Fig. 2E).

#### Data modelling and knowledge transfer

We developed code to model growth dynamics of stigma area and ovary diameter (Fig. 2F). The open-access customisable R scripts (see supplementary Materials and Methods) can produce a range of outputs to compare genotypes, environmental conditions or specific developmental stages, thereby helping to generate new hypotheses. Additionally, the exploitation of the knowledge generated could be key in the progress towards establishing successful hybrid breeding programmes, as the selection of MS cultivars can now be based on previously unreported phenotypic information (Fig. 2G).

### Development and validation of the stigma and ovary convolutional neural networks

**Fig. 3.**
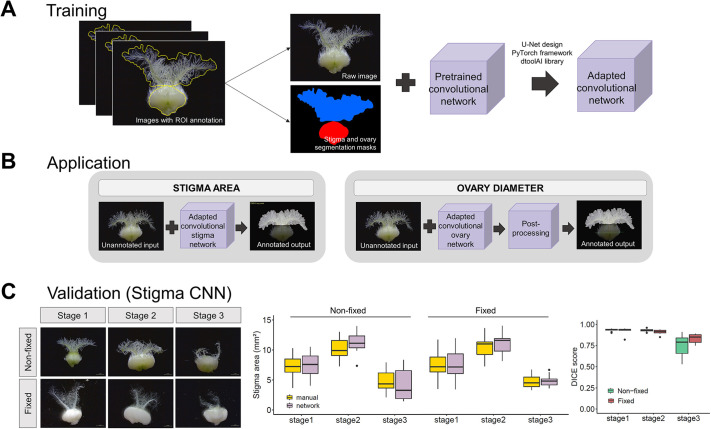
**Development and validation of a CNN for stigma and ovary annotation.** (A) Pipeline for the development of the stigma and ovary networks. Region of interest, ROI. (B) Application of the adapted network for stigma area annotation to new data. (C) Cross-validation of ground-truth measurements and network values extracted from 60 randomly chosen images divided into six classes according to floral age (stage) and sampling method. The first box plot (middle) shows the distribution of the stigma areas in mm^2^ per cross-validation class (*n*=10 images per stage×method combination) determined by manual (yellow) and network (pink) annotation. There were no significant differences for any of the six classes (stage×sampling method combination). The second box plot (right) indicates DICE scores of stigma area in non-fixed (green) and fixed (red) samples. A DICE score of 0 indicates no spatial overlap between the two sets of annotation results, 1 indicates complete overlap. The box plots show the middle 50% of the data with the median represented by the horizontal line. Whiskers represent datapoints within 1.5 times the interquartile range with outliers highlighted as individual points. Scale bars: 1 mm.

Our aim was to develop an automated phenotyping method to detect and annotate the perimeter covered by the stigma hairs and the ovary to determine stigma area and ovary diameter across the life cycle of wheat carpels. To carry out automated image annotation and measurement, we trained a convolutional neural network (CNN) on a set of representative carpel images with manual annotations of the stigma perimeter (*n*=86 images) and ovary perimeter (*n*=121 images) using the UNet design (Falk et al., 2019) (Fig. 3A). The training dataset spanned a random sample of seven genotypes, ranging from early carpel development (W9.5 and earlier) to fully degenerated carpels (Fig. 1A), and included both fixed and non-fixed carpels. After successfully training the networks, we obtained an adapted stigma CNN that is able to quantify the area covered by stigmatic hairs and an adapted ovary CNN that, after some post-processing of the network output, quantifies the diameter of the ovary (Fig. 3B).

To evaluate the performance of the resulting adapted CNNs, we applied the network to an unseen set of 60 microscopy images without manual annotations (Fig. 3C). Subsequently, we manually annotated this new dataset using Fiji to provide a ground-truth reference with which to compare the CNN annotated outputs. We divided the cross-validation process according to the developmental stage of the carpels, sampling method (fixed or non-fixed), and the tissue of interest (Fig. 3C; Fig. S4 for ovary CNN validation). We observed a high overlap in stigma areas and ovary diameters of fixed and non-fixed samples between the manual and automated annotations across all three developmental stages. There were no significant differences between the manual and automated annotations, apart for ovary diameter of the non-fixed samples at stage 3 (*P*=0.03, one-way ANOVA with Tukey's post hoc test; Table S2). Additionally, we calculated the Dice similarity coefficient (DSC) for each group of images that allowed quantitative evaluation of the performance of the adapted networks (Fig. 3C; Fig. S4). Overall, we found very uniform DSC values between the ground-truth and CNN annotation across floral traits, sampling methods and developmental stages (with the exception of the stigma CNN at the last developmental stage), with DSC averages of 0.89 and 0.95 for the stigma and ovary CNNs, respectively. Taken together, these results show that our machine-learning approach quantifies key parameters of the carpel life cycle in wheat, which are in agreement with the more time-consuming manual measurements.

### Variation in stigma and ovary growth patterns can be studied on fixed carpels

Chemical fixation is commonly used to prevent tissue autolysis and degradation, while preserving morphology and cellular details for subsequent macroscopic or microscopic evaluations. Fixatives, however, can lead to changes in the volume and shape of the treated specimens due to cell shrinkage or swelling. Thus, artefacts of the technique could potentially lead to erroneous conclusions when measuring morphological traits of fixed samples.

**Fig. 4.**
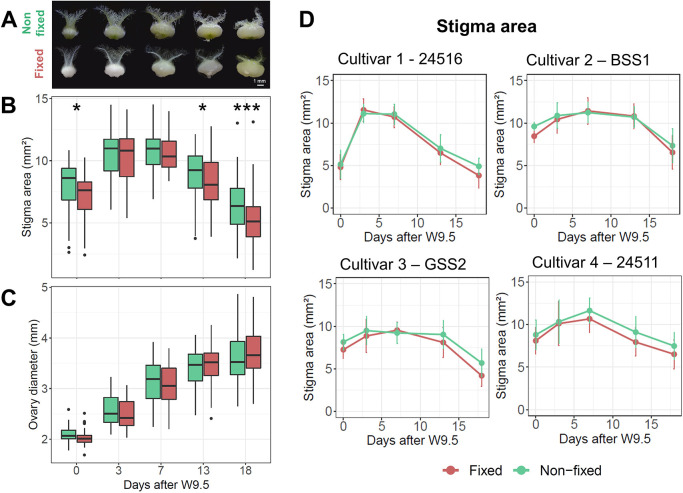
**Effects of the fixative on carpel morphology across time and cultivars.** (A) Representative images of carpels before (non-fixed) and after (fixed) applying the fixative. (B,C) Box plots showing the comparison between non-fixed (green) and fixed (red) samples for stigma area (B) and ovary diameter (C) at different sampling points. Data are the average of the four cultivars shown in panel D, and represent 10-20 carpels from a total of four plants per timepoint and cultivar. *P<0.05; ***P<0.001 (Tukey's test). The box plots show the middle 50% of the data with the median represented by the horizontal line. Whiskers represent datapoints within 1.5 times the interquartile range with outliers highlighted as individual points. (D) Developmental dynamics of stigma area of four MS cultivars, comparing non-fixed (green) and fixed (red) carpel samples (between 10 and 20 carpels from a total of four plants per timepoint). Error bars denote the s.e.m.

To assess the effect of the fixative solution (ethanol and acetic acid) on stigma area and ovary diameter, we used four MS cultivars and sampled carpels at five timepoints over an 18-day period. Analysis of variance (three-way ANOVA) indicated that the fixative significantly reduces stigma area (*P<*0.001), whereas ovary diameter remained unchanged after applying the fixative (*P=*0.25) (Fig. 4A-C; Fig. S5). For stigma area, the fixative×timepoint interaction was borderline non-significant (*P=*0.09) (Table S3), suggesting that the response to the fixative might change with floral age. By analysing individual timepoints, we observed that at 3 and 7 days after W9.5, at the peak of the stigma area, there were no significant differences between fixed and non-fixed samples, whereas at 0 and 13 days (*P*<0.05), and at 18 days (*P*<0.001) after W9.5 (Fig. 4A), the fixed samples show a reduced stigma area. Importantly, the absence of a significant fixative×cultivar interaction (*P>*0.52) suggests that all four cultivars react to the fixative in a similar manner (Fig. 4D). Taken together, we saw that fixing the carpels in ethanol and acetic acid reduced stigma area, even though the developmental dynamics of stigma area were conserved in the four cultivars across the 18 days (Fig. 4D). We therefore conclude that the use of the ethanol and acetic acid fixative allows us to accurately capture the growth dynamics of stigma area and ovary diameter, and to investigate phenotypic variation among diverse genotypes. Nonetheless, caution must be taken to compare absolute stigma areas across development, given the significant reduction at early and late timepoints.

### Application of the phenotyping approach provides insight into the developmental behaviour of the unpollinated wheat carpel

Having established the method to quantitively measure the progression of carpel development in the absence of pollination, we next sought to employ this approach to gain insights into genetic and environmental variation for these two floral traits. To accomplish this, we applied our phenotyping approach to three MS cultivars grown during two consecutive field seasons (2020 and 2021), for which we performed a developmental time course starting from W9.5 until the stage at which the carpel had visually deteriorated. We selected MS cultivars BSS1, 24522 and 24512 as they captured a large part of the variation observed in a broader panel phenotyped in 2020 (M.M.B., unpublished). To accommodate for season-specific differences in temperature between the two seasons (Fig. S2), we incorporated daily temperatures in our model to normalise developmental stages by cumulative degree days.

**Fig. 5.**
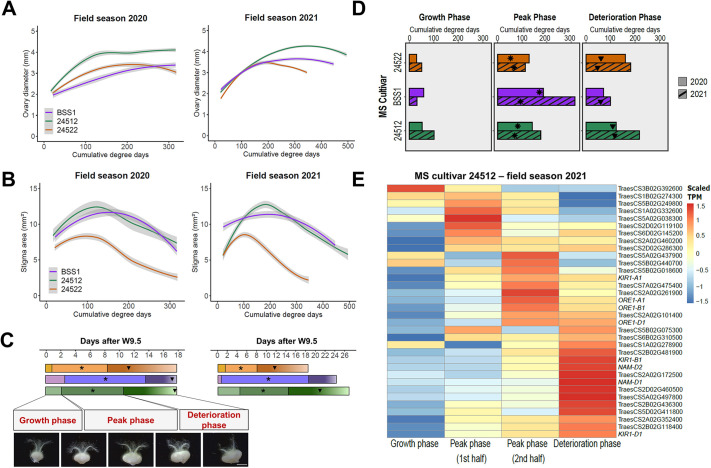
**Phenotypic quantification of carpel development in three distinct MS cultivars under field conditions.** (A) Temporal trends of ovary diameter (mm) in 2020 and 2021. (B) Temporal trends of stigma area (mm^2^) for 2020 and 2021 field seasons. (C) Growth, peak and deterioration phases represented in days after W9.5 for 24522 (orange), BSS1 (purple) and 24512 (green). The temporal units are directly comparable with cumulative degree days shown in panel A. Representative images illustrating carpel appearance at the beginning and end of each phase are shown (the third picture represents a carpel with 100% stigma area). Scale bar: 2 mm. (D) Bar charts show the duration (in degree days) of each of the phases across cultivars and field seasons. The end of the deterioration phase is marked by the last sampling point. In A and B, polynomial regression models at a 95% confidence interval (Loess smooth line) are shown. Grey shading represents the s.e.m. Five carpels from four and six plants were sampled at each timepoint in 2020 and 2021, respectively. In C and D, asterisks indicate maximum stigma area; black triangles indicate a 40% drop in stigma area with respect to the maximum area. Before plotting, outliers were filtered out following the interquartile range criterion. (E) Heatmap illustrating the relative expression of *KIR1*, *ORE1* and genes associated with leaf senescence (Borrill et al., 2019) in stigma samples from MS cultivar 24512. Samples were collected in the growth, peak (first and second half) and deterioration phases during the 2021 field season. Expression levels are normalised within each gene across the four timepoints and rows are sorted according to the similarity of expression and waves of expression in each phase of stigma development. For absolute expression values, see Table S4.

We found that all three MS cultivars exhibited contrasting developmental patterns for stigma area and ovary diameter, and that these differences among cultivars were largely maintained across field seasons (Fig. 5A,B). Phenotypic differences, particularly in stigma area, were observed in both the growth (positive slope) and deterioration (negative slope) phases of carpel development, which inevitably impacted on the overall duration of the life cycle. For instance, we could distinguish the fast development of carpels from cultivar 24522 from the slow progression of carpels from cultivar BSS1 (Fig. 5B). Despite these differences, all three patterns seem to underline a common developmental trend for the dynamics of stigma and ovary traits that is characterised by an initial growth phase, followed by a peak phase in which the stigma reaches its maximum and the radial expansion of the ovary slows down, and a final deterioration phase. This conceptual framework for quantifying and classifying the development of the unpollinated carpel is presented in Fig. 5C,D. The results obtained from breaking down late carpel development into more descriptive phases are detailed below.

#### Growth phase

The stigma and ovaries experienced rapid and exponential growth during the first phase. The growth phase was underway at W9.5 (around ear emergence) and extended for 1 to 4 days until the stigma was well developed and potentially receptive for pollination. The end of the phase coincided with a developmental stage parallel to W10 (anthesis) in male fertile plants. We found that for all three cultivars, the end of this phase could be described by the stigma showing an area of approximately 85% of its maximum area measured. The criteria for us to select the 85% cut-off for stigma area as the end of the growth phase was based on 85% being the percentage that was present in all three cultivars across both field seasons and happened shortly after W9.5, thus mimicking anthesis (Fig. S6).

#### Peak phase

This second phase is denoted by the stigmatic tissue reaching its maximum size (asterisks in Fig. 5C) at around 5 to 10 days after W9.5 (depending on the cultivar and year). After reaching this peak, a gradual and irreversible decline in stigma area was accompanied by a notable arrest of the ovary radial expansion. To mirror the behaviour of stigma area at the beginning of this phase, we selected a 15% drop in stigma area to mark the end of the peak phase. Using this classification, we observed that this phase extended until 8 to 14 days after W9.5 in 2020, and 8 to 18 days in 2021 (Fig. 5C). Considering previous studies on female receptivity in wheat in which the hybrid seed set was maintained from 7 to 13 days (Pickett, 1993; Kirby, 2002; De Vries, 1971), we hypothesise that the peak phase of wheat stigma development coincides with the maximum reproductive potential of the carpel under free pollination conditions. Nonetheless, further analyses of pollen germination and seed setting rates during each of the three phases are needed to prove this hypothesis. Ideally these experiments would be conducted both under free-pollinating conditions (i.e. field trials mimicking hybrid production blocks) and using controlled hand pollinations, as results will most likely differ between experiments owing to the fact that other aspects of the female flower (e.g. opening angle of glumes) play a part in the reproductive potential of the floret (Selva et al., 2020).

#### Deterioration phase

During this phase, symptoms of stigma deterioration started to become obvious and clusters of stigma hairs collapsed in response to a loss in turgor pressure (inverted triangles in Fig. 5C indicate a 40% drop in stigma area). The collapse of the remaining stigma hairs continued for several days, resulting in a completely deteriorated stigma at 18 and 27 days after W9.5 in 2020 and 2021, respectively (Fig. 5C,D). By the end of this phase, the ovary walls also showed an irregular surface due to tissue deterioration. Based on these observations, we speculate that the onset of this phase marks the irrevocable loss of the reproductive potential of the floret.

Dissecting carpel development into growth, peak and deterioration phases allowed us to assign the cultivars BSS1, 24512 and 24522 into slow-, moderate- and fast-developing carpels, respectively, according to when they reached the beginning of the deterioration phase. For example, cultivar BSS1 reached the onset of the deterioration phase at 14 and 18 days after W9.5 in 2020 and 2021, respectively. This was approximately 4 days after the onset of the deterioration phase for cultivar 24512, and 6 and 10 days after the onset of the deterioration phase for cultivar 24522 in 2020 and 2021, respectively (Fig. 5C). The developmental pattern classification also allowed comparisons across field seasons, for which the relative ranking of cultivars was well conserved between years. Although we adjusted for cumulative degree days, the colder and damper weather conditions of 2021 (Fig. S2) were reflected in an extension in the duration of most phases in some but not all cultivars (Fig. 5D). For example, the durations of the three phases were largely unaffected in cultivar 24522, whereas in cultivar BSS1, the duration of the peak phase in 2021 (315 degree days) was almost twice that of the previous year (186 degree days). These initial analyses suggest that although the developmental dynamics of stigma area and ovary diameter were largely consistent across years, there were cultivars in which the duration of the developmental phases could be sensitive to other environmental changes that remain to be tested.

To provide further support for the proposed stages of the stigma life cycle, we investigated the expression of genes encoding senescence-related transcriptional regulators that we expect to be upregulated during the late peak and deterioration phases. Stigmas were sampled across the different phases of development using field-grown MS cultivar 24512, and the extracted RNA from these samples was used for an RNA-sequencing transcriptome experiment. We focused on the expression of wheat homologues of the *KIR1* and *ORE1* transcription factors (TFs) (Fig. S7), shown previously to promote stigma senescence in *Arabidopsis* (Gao et al., 2018), and the 36 top-ranked genes proposed to coordinate leaf senescence in wheat (Borrill et al., 2019), which included genes belonging to the GRAS, HSF and RWP-RK TF families. All six *KIR1* and *ORE1* wheat homologs were expressed, and 28 of the 36 TFs associated with leaf senescence were also expressed in the stigma, suggesting a large overlap between leaf and stigma senescence processes (Fig. 5E). Of the 34 TFs expressed in the stigma, we identified distinct waves of expression in their transcript profiles across the four sampling timepoints. Although eight TFs had the highest expression in the growth and early peak phase, over 70% of the expressed senescence-related TFs (including wheat *KIR1* and *ORE1*) were upregulated towards the end of the peak phase (ten genes) or at the deterioration phase (15 genes) (Fig. 5E). These results highlight that the proposed phases of late carpel development, determined using microscopy, associate with expected transcriptional changes based on the biological processes that occur during these later stages (Fig. 5E).

## DISCUSSION

### High-throughput phenotyping for the quantification of floral traits in unpollinated wheat carpels

Our understanding of floral developmental processes has been assisted by the establishment of scales that describe changes in the shape, size and surface features of floral organs leading up to anthesis (Waddington et al., 1983; Smyth et al., 1990). These scales often divide a continuous developmental process into defined stages, which are characterised by landmark events, such as the appearance of stigmatic branches. These scales have facilitated the interpretation of genetic studies and have contributed towards our understanding of the mechanisms that underlie the transitions leading to a given landmark event. During post-pollination stages, the focus shifts towards the developing fruit. Previous work, however, has also illustrated the importance of the quantitative monitoring of morphological changes associated with late carpel development (i.e. in the absence of pollination), such as silk elongation in maize (Bassetti and Westgate, 1993) or ovary radial expansion in wheat (Okada et al., 2018), as they represent survival mechanisms to ensure seed set by cross-pollination. So far, the few studies investigating the progression of the unpollinated female carpel after anthesis have focused in giving detailed descriptions of flowers from one or two different genotypes grown in controlled environment conditions. These types of meticulous approaches are arduous and expensive not only to implement in large-scale experiments but also to execute under field conditions, in which equipment is often limited. Consequently, studies investigating detailed phenotypes in the field are lacking.

To enhance our understanding of the biological processes that occur in the unpollinated carpel under breeding-relevant conditions, we created a machine-learning-based approach to phenotype field-grown MS wheat cultivars. Given the sequence of morphological changes that we observed in the unpollinated wheat carpel (Fig. 1A), we quantified changes in stigma area and ovary diameter to describe carpel development. Our findings are twofold: (1) we demonstrate the suitability of our approach for the detailed study of floral organ traits in field screenings, and (2) we show that the unpollinated carpel undergoes a well-defined pattern of growth and senescence characterised by gradual changes in stigma and ovary sizes (Fig. 5A,B). Based on these findings, we propose developmental phases that are relative to the maximum stigma area and ovary size (Fig. 1C, Fig. 5C), and with which to build the foundations of future research of floral organ development and senescence in the absence of pollination.

### Considerations on the use of the stigma- and ovary-adapted CNNs

The quantification of stigma area and ovary diameter cannot be easily determined from surface observations of wheat spikes and requires the dissection and microscopy of individual carpels. The manual annotation and quantification of microscopy images is cumbersome and often delays scientific discoveries. Deep-learning-based approaches, such as the use of CNNs, have emerged as a solution to perform image quantification in an automated, rapid, and less biased manner, lifting the burden of image analysis from researchers. In this work, we developed two CNNs (both publicly available at https://github.com/Uauy-Lab/ML-carpel_traits) that enable non-machine-learning experts to quantify stigma area and ovary diameter on their local computer in a matter of hours (Fig. 3). Evaluation metrics on the performance of the adapted networks (Fig. 3C; Table S2) demonstrate their capability to satisfactorily measure both floral traits by condensing each RGB image to a single value (i.e. pixels). Measurements between manual and CNN annotation were largely indistinguishable (Table S1), with the network being less capable at later stages (Fig. 3C; Fig. S1). We believe that one of the reasons for the poorer performance could be due to the difficulty in distinguishing the stigma and ovary from each other when the stigma is severely deteriorated and resting on top of the ovary (Fig. S8). Also, it is worth noting that the ovary CNN relies on the performance of the stigma CNN to correctly predict the diameter of the ovary (see Materials and Methods for the detailed description of the algorithm), such that a bad prediction of the stigma area will likely affect ovary annotation. Poor-quality images (i.e. out-of-focus or poor-resolution images) and certain carpel orientations (Fig. S3) also hinder the identification of the ovary and/or stigma, impairing the normal performance of the CNNs. In the case of blurry images, using post-processing tools (e.g. Photoshop or other image-sharpening techniques) to adjust the sharpness of the image might reduce the likelihood of incorrect annotations. However, there will still be certain cases for which there may not be an immediate reason for the failure of the CNN. We therefore suggest including an additional output-verification step (Fig. 2E) to identify potential errors before continuing with the downstream analyses.

### The implementation of the phenotyping approach opens new research paths on the biology of late carpel development

To gain a more comprehensive overview of the developmental dynamics of the unpollinated carpel, we used our phenotyping approach (Fig. 2) to examine the sequential progression of changes in stigma and ovary morphology in three MS cultivars over two field seasons (Fig. 5). Across cultivars and seasons, we were able to identify an initial stigma and ovary growth phase, followed by a peak phase describing carpel developmental maturity, and a subsequent deterioration phase characterised by the eventual collapse of the female reproductive tissues and expression of multiple senescence-associated genes (Fig. 5C,E). Equivalent patterns for the post-anthesis development of the unpollinated stigma have also been reported in maize, peas (*Pisum sativum*) and *Arabidopsis* (Bassetti and Westgate, 1993; Vercher et al., 1984; Carbonell-Bejerano et al., 2010), suggesting a conserved developmental programme that ensues in the absence of pollination.

Despite the conserved overall patterns, we identified differences in the duration of the growth, peak and deterioration phases in the three wheat MS cultivars used here. Gene expression studies of unpollinated carpels and stigma in *Arabidopsis* and maize (Carbonell-Bejerano et al., 2010; Gao et al., 2018; Šimášková et al., 2022) have demonstrated that the lifespans of these floral structures are controlled by transcription factors that regulate developmental programmed cell death in these tissues. Here, we also identified distinct waves of expression of genes encoding senescence-associated TFs across late stigma development in a single wheat MS cultivar. Our results, along with previous evidence from *Arabidopsis* and maize, raise the prospect that the phenotypic variation observed among the three wheat MS cultivars could be due to differential gene expression patterns across cultivars that alter the onset of stigma senescence (Fig. 5B,C). Thus, new transcriptomic studies investigating the developmental transitions observed among the different field-grown cultivars would contribute to our understanding of the mechanisms governing these phases. We also observed that MS cultivars 24512 and 24522 had largely equivalent peak phase durations across years, whereas the duration of the peak phase in the CMS cultivar BSS1 varied almost twofold between field seasons (Fig. 5D). This suggests that, despite accounting for temperature in our analyses (by using cumulative degree days), the duration of the stigma peak phase is sensitive to additional environmental factors. The response to these additional environmental factors could depend on the genotype, sterility system used and/or the developmental phase in which the environmental stimuli are encountered. Consistent with this, several studies in wheat have attributed differential seed set rates of out-crossing MS plants (i.e. an indicator for the duration of stigma receptivity) to environmental factors such as temperature, relative humidity and soil water availability (Fábián et al., 2019; De Vries, 1971; Imrie, 1966). Therefore, additional studies under field and controlled environment conditions will shed light on the causalities for the variation observed in ovary and stigma development across field seasons. Our phenotyping approach now improves the accessibility of the wheat carpel to detailed phenotypic analyses of the size of populations that are used in breeding programmes. This facilitates the identification of mutations that underpin genetic variation in carpel development, thus contributing to understanding gene function on a genome-wide scale. All in all, we provide a framework in which to conduct these new studies targeting diverse environments and genotypes, which will facilitate future hypothesis generation not only in wheat but also in other cereal crops.

### First steps towards an integrated developmental scale of the unpollinated wheat flower

The ultimate role of the carpel is the production of a viable seed. Thus, increasing the functional lifespan of carpels and stigmas (i.e. floral receptivity) are desirable agronomic traits that have the potential to increase the effective pollination period and seed set (Williams, 1965). Yet, detailed evaluations of carpel and stigma development and how they relate to female floral receptivity and seed set are still lacking, especially in cereals. It is reasonable to think that the functional lifespan of stigma receptivity would coincide with stigma cell integrity, as illustrated in early studies of kiwifruit (*Actinidia deliciosa*) and maize (González et al., 1995; Bassetti and Westgate, 1993). According to these studies, we could, for example, speculate (1) that seed set rates will be higher if pollination occurs during the peak phase compared with the deterioration phase, or (2) that cultivars with a prolonged peak phase (such as BSS1) will be receptive to pollination for longer than cultivars with a shorter peak phase (such as 24512 or 24522) (Fig. 5C). However, as recently demonstrated in *Arabidopsis*, a delay in stigma senescence caused by the disruption of two transcription factors promoting programmed cell death was only accompanied by a minor extension in floral receptivity, suggesting that additional processes must be involved in controlling the duration of floral receptivity, for instance, ovule viability (Gao et al., 2018). New studies, therefore, need to be conducted to investigate stigma receptivity under defined phases of carpel development to clarify the relationship between stigma morphology and viability, pollen germination, and seed set. Additionally, such information will allow a greater understanding of how genetic and environmental factors affect various aspects of the stigma life cycle (e.g. loss of stigma receptivity, onset of stigma cell death). The next steps towards understanding the cross-pollination process in the field will also require integrating the changes in carpel morphology with those of the overall spike. For instance, as reviewed by Selva et al. (2020), certain wheat spike architectures, like the openness of the floret, facilitate airborne pollen access, which would additionally contribute to increasing out-crossing rates in hybrid production.

Our approach for phenotyping carpel development provides a new tool for examining a fertility trait that is poorly understood and hitherto time consuming to analyse. Taken together with recent advances in genetic resources (Krasileva et al., 2017; Wingen et al., 2014; Sansaloni et al., 2020) and genome sequence data (Walkowiak et al., 2020; International Wheat Genome Sequencing et al., 2018), this approach provides a new opportunity to unlock genetic variation for stigma and ovary traits that associate with floret fertility, which is vital given that improved fertilisation will help in addressing the increasing demands to enhance global food production.

## MATERIALS AND METHODS

### Germplasm

We used both spring and winter MS hexaploid wheat (*Triticum aestivum*) cultivars derived from commercial inbred lines. CMS and NMS systems were used for the generation of the male sterile cultivars. BSS1 and GSS2 correspond to winter CMS cultivars, whereas the winter cultivars 24511, 24512, 24516 and 24522, and the spring cultivars Jetstream, Alderon, BLA1, Mairra, Cadenza, Chamsin and BLA2 are NMS cultivars. CMS and NMS cultivars were provided by KWS (Thriplow, UK) and Syngenta (Whittlesford, UK), respectively.

We used MS cultivar 24516 as an example to illustrate the developmental dynamics of the unpollinated wheat stigma and ovary in Fig. 1. To train the CNN for the quantification of carpel traits, we used a random sample of plants extracted from a set of the seven spring NMS cultivars. The selection criteria for the generation of the training set, however, were based on the diversity of carpel images rather than being based on individual cultivars.

We used four winter MS cultivars (24511, 24516, BSS1 and GSS2) to characterise the effects of the fixative on stigma area and ovary diameter during the 2020 field season. Finally, for the multi-year field experiment performed to investigate the developmental patterns of the unpollinated carpel, we grew three winter MS cultivars (BSS1, 24512 and 24522) during two consecutive years. We selected these three cultivars as they represent a large part of the variation observed for carpel development in the absence of pollination from an original pool of 31 MS cultivars phenotyped in 2020 under field conditions (M.M.B., unpublished). Table S1 provides a summary of all cultivars used in the experiments.

### Glasshouse and field experiments

For the development of the stigma and ovary CNNs, we grew between ten and 20 plants per MS cultivar (Jetstream, Alderon, BLA1, Mairra, Cadenza, Chamsin and BLA2) in the glasshouse in 1 l pots under long day conditions (16 h light: 8 h dark) and hand dissected carpels from either the first, second or third tiller at various timepoints that were representative of the different carpel morphologies. We stored a random selection of the dissected carpels in a fixative solution of 95% ethanol and absolute acetic acid (75% v/v), and kept them at 4°C until image acquisition (approximately 1 month after fixation). The cultivars selected are representative of carpel morphology diversity in wheat germplasm.

For the carpel development time course experiments, we grew plants at John Innes Centre Church Farm (Bawburgh, UK; 52°37′50.7″N 1°10′39.7″E) in a randomised complete block design with two replicates (1 m plots) per MS cultivar in 2020, and three replicates in 2021 (Fig. S1). The number of replicates were chosen according to traditional use in field experiments and seed availability. To avoid unwanted cross-pollination, sterile rye barriers were grown surrounding the MS plots. To record environmental data, we placed data loggers (EasyLog USB, Lascar Electronics) next to the experimental plots at a 50 cm height. Average temperature and relative humidity were measured every hour for the duration of the experiment (Fig. S2).

In both field seasons, we tagged main spikes when carpels in the outer florets (floret 1 and 2) of central spikelets reached W9.5 (Waddington et al., 1983). This is shortly after full ear emergence (Zadoks growth stage 59; Zadoks et al., 1974). At the time of sampling, we cut individual tillers between the uppermost and penultimate internode and transported them in water to the laboratory for carpel dissection. We harvested four to seven carpels from the outer florets of central spikelets from two spikes per plot and timepoint. These timepoints were W9.5 and 3, 7, 13 and 18 days after W9.5 (DAW9.5), with the only exception that the 2021 time course was extended for nine more days (i.e. 27 DAW9.5) in cases in which the carpel was not completely senesced at 18 DAW9.5. Owing to the limited availability of spikes tagged at W9.5, sample sizes for the extended timepoints varied from two to six spikes per timepoint. Carpels that needed fixation were placed in 2 ml Eppendorf tubes containing fresh fixative solution, as described above.

### Image acquisition and manual annotations

To generate the training set, we used two different stereo microscopes equipped with an integrated camera for image capture (ZEISS Stemi 305 with a 1.2 Megapixel integrated colour camera; Leica MZ16 coupled with a Leica CLS100x white light source; and a Leica DFC420 5 Megapixel colour camera). For the downstream experiments using the adapted CNNs, we only used the ZEISS Stemi 305, as it is easier to operate and to transport to the field. Depending on the size of the carpel, we used different magnifications (from 1× to 4×) to ensure that the complete carpel was captured in the image (Fig. S3). We adapted the illumination and time of exposure for each image to ensure high contrast between the carpel and the black background. To maintain the feathery structure of the stigma in fixed samples, we imaged the carpels submerged in a 70% ethanol solution using a Petri dish (one carpel per image). Images were saved as RGB JPEG files.

To evaluate the effect of the fixative on carpel morphology traits, we first imaged the carpels as non-fixed samples and then placed them in the fixative solution (as described previously) for image acquisition at a later time. For manual annotations of stigma and ovary perimeters, we analysed the resulting images using the open-source image-processing package Fiji.

### Development of stigma and ovary CNNs

To carry out automated image annotation and measurement, we trained a CNN using the U-Net design, which can be easily adapted to new tasks with only few annotated images (Falk et al., 2019). The network was implemented in the PyTorch framework (Paszke et al., 2019), using the dtoolAI library (Hartley and Olsson, 2020). The networks were trained on carpel JPEG images with manual annotations of the stigma perimeter (*n*=86 images) and ovary perimeters (*n*=121 images). These 207 images were randomly selected from a total of 1601 dissected carpels. The Dice coefficient (Dice, 1945) was used as a loss function, and weight updates were applied using the Adam optimizer (Kingma and Ba, 2015).

The trained stigma network predicted a mask corresponding to the stigma location for each RGB input image. The stigma mask was used to calculate the stigma area directly by extracting the number of pixels. The trained ovary network predicted two masks for each RGB input image: one corresponding to the ovary location and the other to the stigma location. To determine the ovary diameter, the following algorithm was applied: (1) determination of the centroid of the predicted stigma mask; (2) determination of the centroid of the predicted ovary mask; (3) taking the perpendicular to the line drawn through the centroids; (4) determination of the two points where this line crosses the border of the predicted ovary mask; and (5) measurement of the length of this line, converting to physical units from the original input image scale.

We converted pixels (CNN output) to stigma area (mm^2^), and ovary diameter (mm) according to the scale bar used in each image. Implementation scripts and training data are available at https://github.com/Uauy-Lab/ML-carpel_traits and https://opendata.earlham.ac.uk/wheat/under_license/toronto/Millan-Blanquez_etal_2022_machine-learning-carpel-traits/, respectively.

### Statistical analyses and data visualisation

#### Evaluation of CNN performance

To evaluate differences in stigma area and ovary diameter between the manual and CNN annotations, we selected a random set of 60 microscopy images that were not used to train the networks (30 images of fixed carpels and 30 of non-fixed carpels). We divided the images into three different developmental stages based on the appearance of the carpel to account for all the possible carpel morphologies to which the algorithm could be exposed. Stage 1 represents a young carpel (stigma and ovary still developing), stage 2 represents a fully developed carpel (widely spread stigma and enlarged ovary) and stage 3 includes visibly deteriorated carpels. We performed one-way ANOVA using Tukey's post hoc test for each trait and sampling method, including ‘floral age’ as the single factor (Table S2). To measure the spatial overlap between the manual and CNN annotation, we calculated Dice similarity coefficients on the same set of images.

#### Carpel development time courses

For the quantification of stigma area and ovary diameter of fixed and non-fixed samples, a total of 666 and 634 images were annotated by the stigma CNN and ovary CNN, respectively, and used for subsequent analyses. We conducted three-way ANOVAs (fixative, timepoint and cultivar) to test the effect of the fixative on stigma area and ovary diameter, and their interaction with floral age (timepoint) and cultivar (Table S3). We included block and spike information in the model as random effects to account for the nested nature of the experimental design. Tukey's multiple comparison method was used to adjust for multiple comparisons.

To generate the patterns describing stigma and ovary development for MS cultivars 24512, 24522 and BSS1, 294 images were annotated by the stigma and ovary CNNs in 2020, and 520 images in 2021, and used for downstream analyses. Next, we filtered outliers following the interquartile range criterion and used Loess smooth lines with a span value of 0.9 (i.e. width of the moving window when smoothing the data) and a 95% confidence interval. To have an estimate of the amount of growth the plants achieved during the different field seasons, we calculated cumulative degree days using 0°C as base temperature (according to Miller et al., 2001) and average daily temperatures as follows:

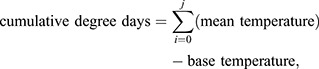
where *i* is the beginning of the temporal window considered (e.g. the W9.5 date) and *j* is the end of the temporal window considered (e.g. sampling date at 7 days after W9.5).

Codes to model carpel and ovary development were executed in RStudio version 4.0.3 and are available at https://github.com/Uauy-Lab/ML-carpel_traits. Image data used for the statistical analyses (i.e. one-way and three-way ANOVAs provided in Tables S2 and S3, respectively) are also available at https://opendata.earlham.ac.uk/wheat/under_license/toronto/Millan-Blanquez_etal_2022_machine-learning-carpel-traits/. Detailed step-by-step instructions are provided in the supplementary Materials and Methods.

### Stigma transcriptome analysis by RNA sequencing

During the 2021 field season, we used MS cultivar 24512 to investigate the expression patterns of senescence-related genes in the stigmatic tissues. We collected stigma samples at four developmental timepoints spanning the proposed growth, peak and deterioration phases (3, 7, 13 and 18 DAW9.5). We sampled plants between 11.00 h and 15.00 h and dissected five to eight stigmas (size dependent) from the primary and secondary florets of the central four spikelets. We stored stigmas in DNA/RNA Shield solution (Zymo Research, R1100-50) at −20°C. The stigma samples included both the stigma and style, as these structures are intimately linked in wheat.

We extracted RNA from three independent biological replicates using TRIzol/Chloroform (TRI Reagent, Sigma-Aldrich; Chloroform, ≥99.8%, Thermo Fisher Scientific) and purified using the RNA Clean and Concentrator kit (Zymo Research, R1013) as specified in the protocol for RNA extraction from wheat stigmas stored in DNA/RNA shield (https://dx.doi.org/10.17504/protocols.io.36wgq7kj3vk5/v1). RNA quantity and quality were assessed by spectrophotometric analysis (NanoDrop One/OneC; Thermo Fisher Scientific) and by agarose gel electrophoresis. Total RNA samples with a quality value greater than an RNA integrity number of 6 were used for Illumina HiSeq 150-bp paired-end sequencing (Novogene).

RNA sequencing data were pseudoaligned to the wheat RefSeqv1.1 transcriptome (International Wheat Genome Sequencing et al., 2018) using kallisto (Bray et al., 2016). We filtered for genes that expressed on average >0.5 transcripts per million (TPM) in at least one of the timepoints to exclude genes with low expression (consistent with Ramírez-González et al., 2018). TPM values were normalised across the four timepoints for each gene, and the individual TPM values are presented in Table S4.

## Supplementary Material

10.1242/develop.200889_sup1 Supplementary information
