# Supplementary material for: A scalable phenotyping approach for female floral organ development and senescence in the absence of pollination in wheat
Source: Development. 2022 Sep 15;149(18):dev200889. doi: 10.1242/dev.200889 (PMC9573784; doi:10.1242/dev.200889)
Supplement: Supplementary information [file develop-149-200889-s1.pdf]

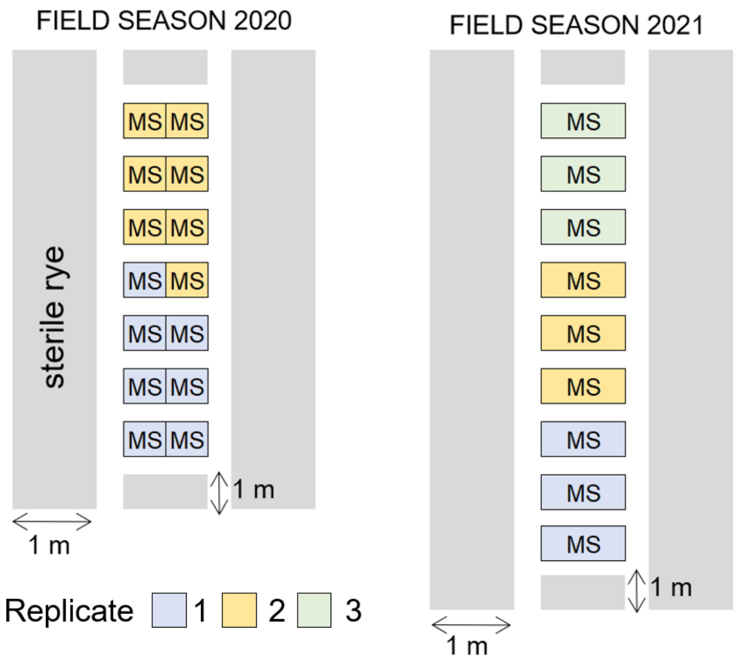

Fig. S1. Schematic representations of the field layout. Male sterile (MS) cultivars were grown surrounded by a continuous stripe of sterile rye that was used as pollen barrier. Plots were replicated twice in 2020 (*N* = 7) and 3 times in 2021 (*N* = 3).

Development • Supplementary information

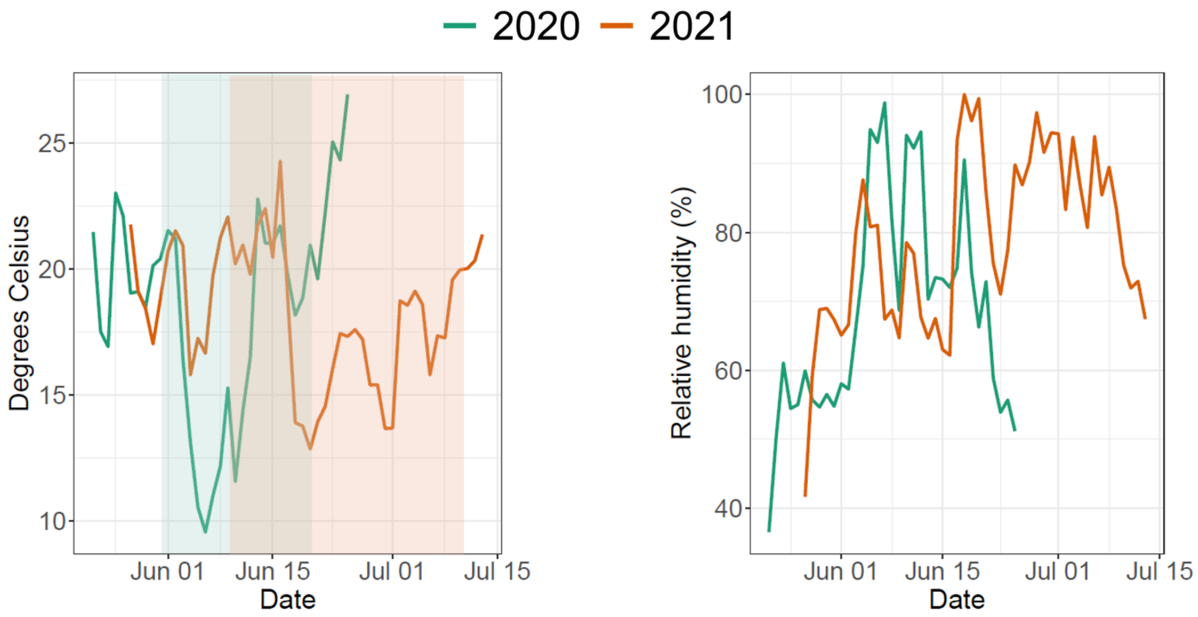

Fig. S2. Environmental conditions recorded during 2020 and 2021 field seasons. Left panel illustrates daily temperatures in degrees Celsius recorded during both field experiments (2020: green line; 2021: orange line). Shaded rectangles indicate the beginning and end of 2020 and 2021 time courses. Right panel shows the water vapor contained in the air expressed in percentage of relative humidity.

Development • Supplementary information

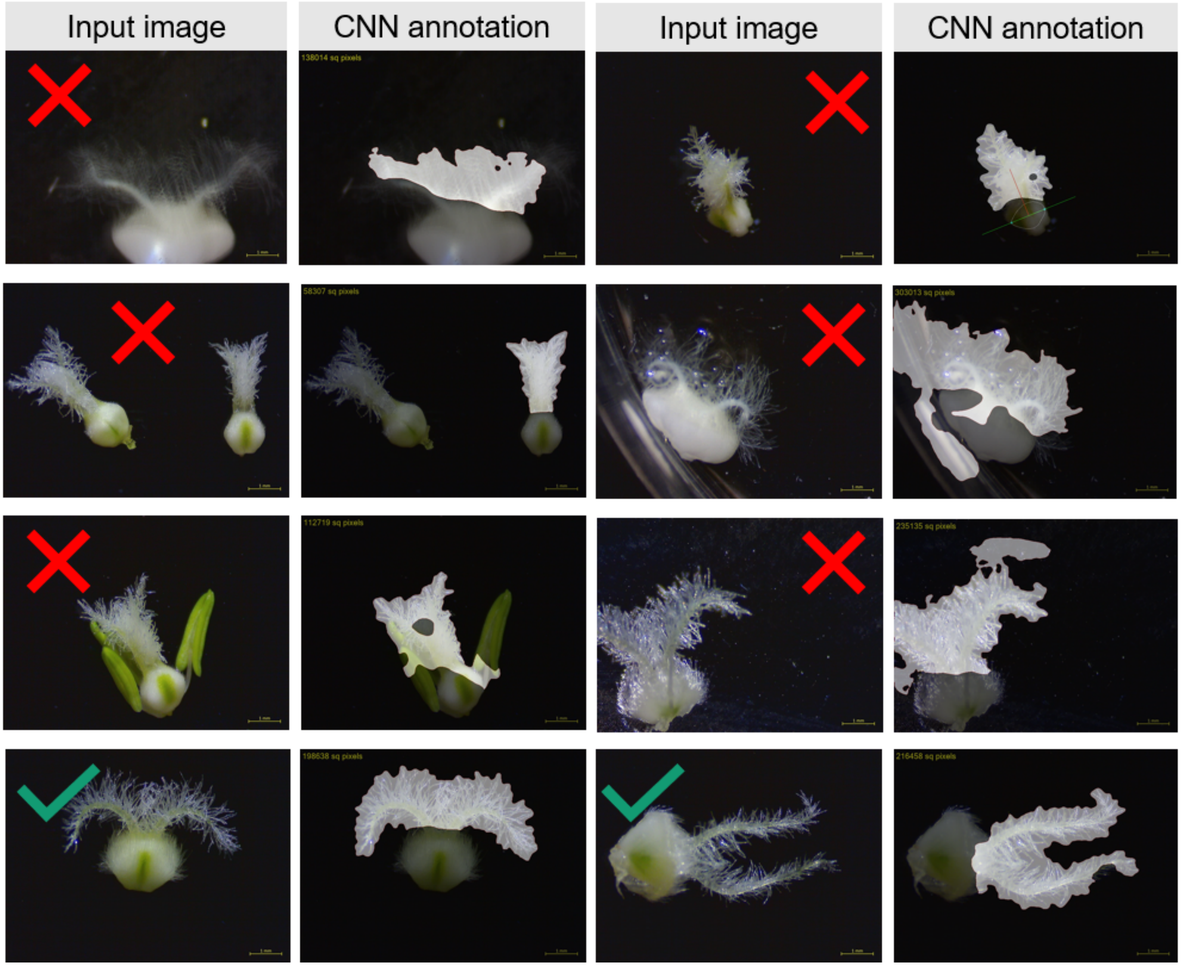

Fig. S3. Unaccepted and accepted carpel images. Representation of images (jpeg format) unlikely to be correctly annotated by either the stigma CNN or ovary CNN (red crosses, e.g., blurry or out of focus images, lifted carpels, presence of other objects, etc.) and example of images the stigma and ovary CNNs expect and would be able to annotate accurately (green ticks).

Development • Supplementary information

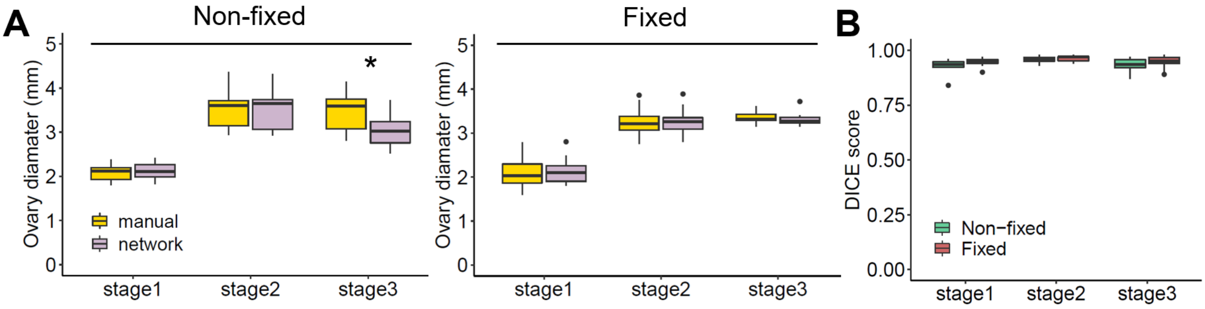

Fig. S4. Validation of convolutional neural network for stigma and ovary annotation. **(A)** Cross-validation of ground-truth measurements and network values extracted from 60 randomly chosen images divided into six classes according to floral age and sampling method. Distribution of the ovary diameter in mm per cross-validation class (*n* = 7-10), determined by manual (yellow) and automated (violet) annotation. **(B)** Box plots showing Dice similarity coefficient of ovary diameter in non-fixed (green; *n* = 10) and fixed (red; *n* = 10) samples (0 indicates no spatial overlap between the two sets of annotation results, 1 indicates complete overlap). Box plots show the middle 50% of the data with the median represented by the horizontal line. Whisker represents datapoint within 1.5 times the interquartile range with outliers highlighted as individual.

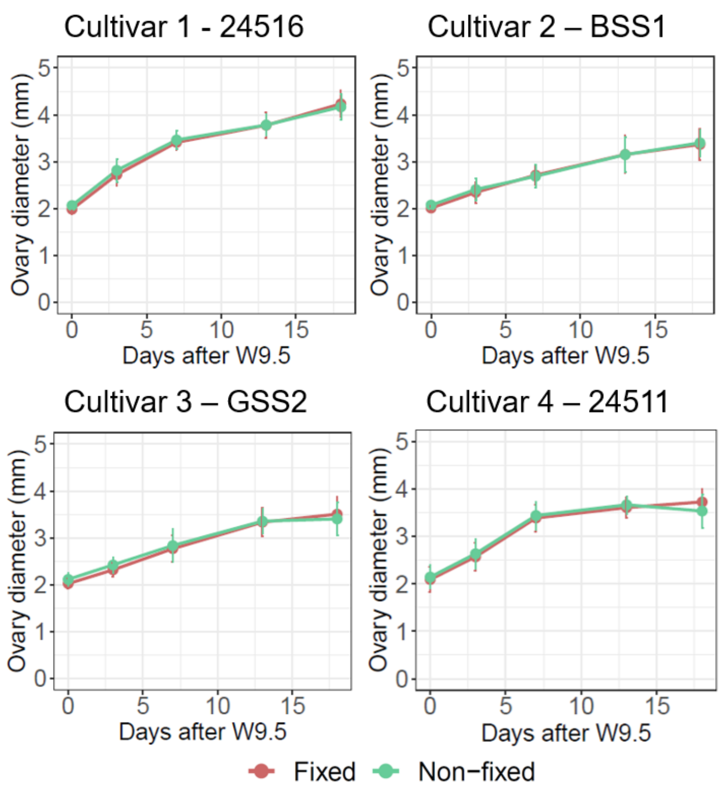

Fig. S5. Effects of the fixative on ovary diameter across time and cultivars. Stigma area development dynamics of four MS cultivars comparing non-fixed (green) and fixed (red) carpel samples (between 10-20 carpels from 4 plants per timepoint). Error bar denotes the standard error.

Development • Supplementary information

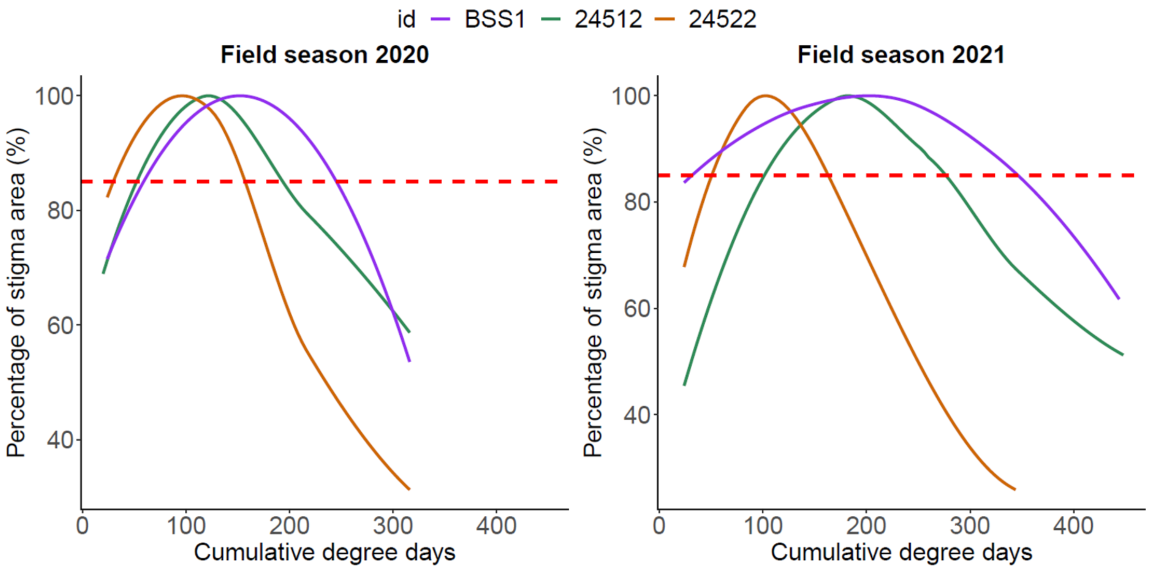

Fig. S6. Developmental stigma patterns expressed in percentage from maximum observed stigma area. Red dashed line indicates 85% benchmark for the selection of the boundaries of the peak phase with the growth and deterioration phases.

Development • Supplementary information

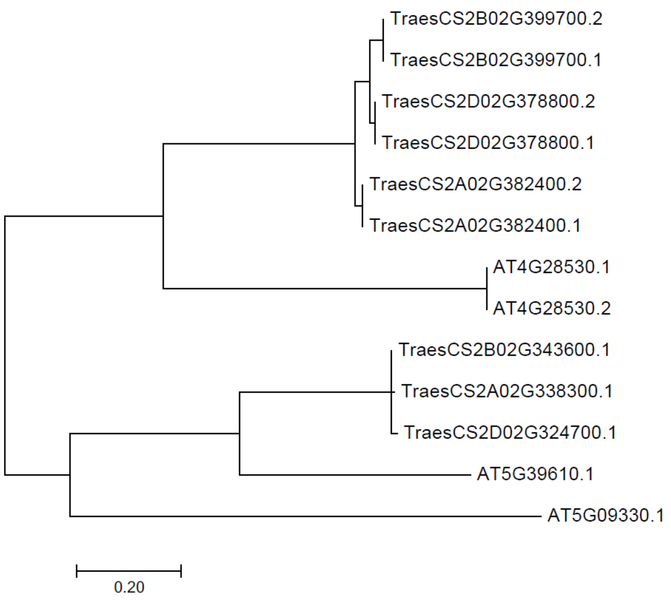

Fig. S7. Phylogenetic tree of *KIR1* and *ORE1* from Arabidopsis and its closest wheat homologs. Peptide sequences used to construct a rooted neighbor-joining tree of both splice variants of *KIR1* (AT4G28530), *ORE1* (AT5G39610) and its orthologs in wheat. *ANAC082* (AT5G09330) was used as outgroup.

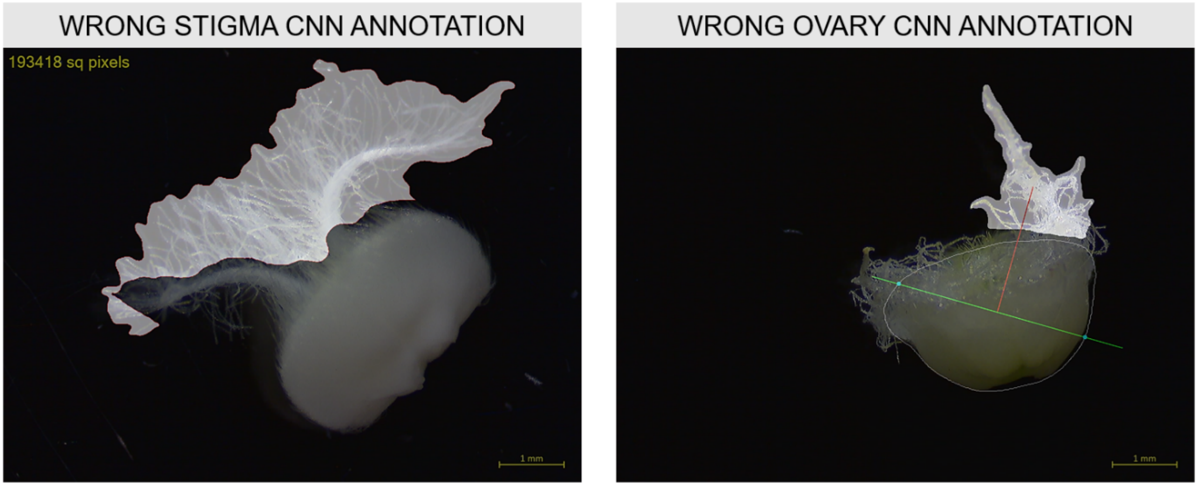

Fig. S8. Erroneous stigma area and ovary diameter CNN annotations. Examples of bright field images of carpels wrongly annotated by the stigma (left) and ovary (right) CNNs.

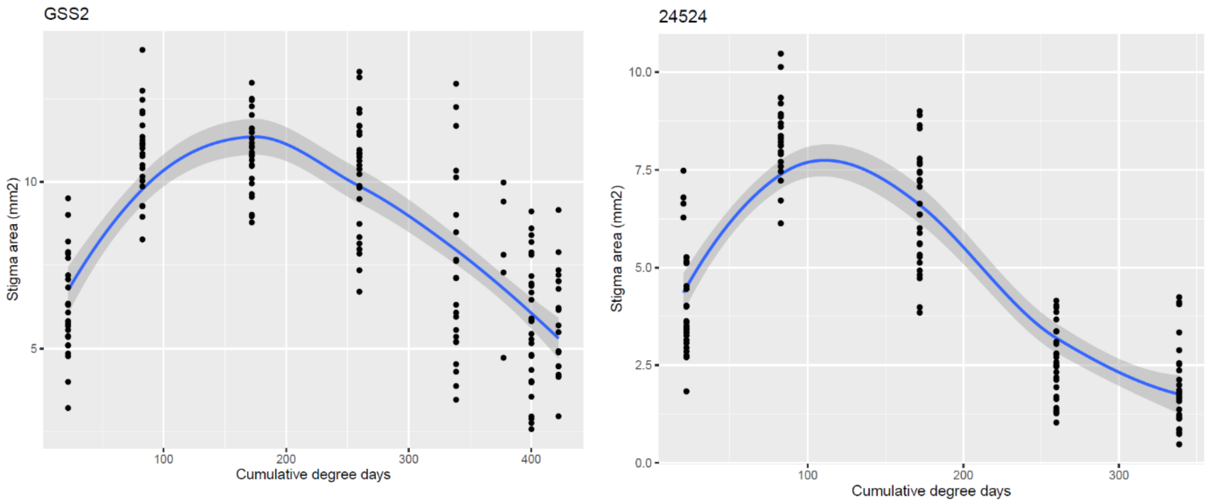

Fig. S9. Visual output obtained after running "Stigma_area_script.R" on the examples provided. Stigma area developmental patterns observed for the two examples provided, GSS2 and 24524. Each dot represents the area of the stigma of an unpollinated carpel. The blue line is a loess smooth line (polynomial regression) and the grey shading indicates the standard error.

Table S1. Summary of the germplasm used for each experiment described in this study.

Experiment	Output	Germplasm used	Growth
Representative stigma and ovary growth patterns	Fig. 1	24516	Field
Training set for the development of stigma and ovary CNNs	Trained CNNs	Jetstream, Alderon, BLA1, Mairra, Cadenza, Chamsin, BLA2	Glasshouse
Effect of fixative on stigma area and ovary diameter	Fig. 4 and S5	24511, 24516, BSS1, GSS2	Field
Multi-year field experiment	Fig. 5	BSS1, 24512, 24522	Field
Stigma transcriptome	Fig. 5E	24512	Field
Mock example provided to run "Stigma_area_script.R"	Fig. S8	24524, GSS2	Field

Table S2. Summary table on the manual and CNN annotation metrics obtained for stigma area and ovary diameter.

	***Stigma area* (mm^2)**								
	**Stage-1**			**Stage-2**			**Stage-3**		
	Manual annot.	CNN annot.	ANOVA *P value*	Manual annot.	CNN annot.	ANOVA *P value*	Manual annot.	CNN annot.	ANOVA *P value*
**Non-fixed carpels**	7.27 ± 0.648	7.54 ± 0.648	0.7741	10.23 ± 0.648	11.16 ± 0.648	0.3118	4.75 ± 0.648	4.19 ± 0.648	0.5488
**Fixed carpels**	7.54 ± 0.605	7.6 ± 0.605	0.9358	10.54 ± 0.605	11.1 ± 0.605	0.514	4.73 ± 0.605	4.84 ± 0.605	0.9005

	***Ovary diameter* (mm)**								
	**Stage-1**			**Stage-2**			**Stage-3**		
	Manual annot.	CNN annot.	ANOVA *P value*	Manual annot.	CNN annot.	ANOVA *P value*	Manual annot.	CNN annot.	ANOVA *P value*
**Non-fixed carpels**	2.09 ± 0.122	2.12 ± 0.122	0.8556	3.58 ± 0.129	3.59 ± 0.129	0.9769	3.48 ± 0.137	3.04 ± 0.137	0.0298
**Fixed carpels**	2.1 ± 0.0975	2.15 ± 0.0975	0.7473	3.27 ± 0.1027	3.27 ± 0.1027	0.9966	3.36 ± 0.1165	3.33 ± 0.1165	0.8659

Note: Estimated marginal means ± standard errors are given for each type of annotation. *P* values indicate statistical significance of the pairwise comparison in one-way ANOVA.

Table S3. Summary table of three-way ANOVA on the effect of the fixative on stigma area and ovary diameter across different timepoints.

	ANOVA *P value*			
	Fixative	Fixative × Timepoint	Fixative × Cultivar	Fixative × Timepoint ×Cultivar
**Stigma area**	6.39E-06	0.08956	0.52333	0.781
**Ovary diameter**	0.24692	0.08693	0.96446	0.98034

	**0 DAW9.5**			
	Fixed	Non-fixed	Fixative effect (%)	*P value*
**Stigma area (mm2)**	7.15 ± 0.265	7.92 ± 0.265	-9.72	0.0292
**Ovary diameter (mm)**	2.01 ± 0.0468	2.09 ± 0.0468	-3.83	0.2299

	**3 DAW9.5**			
	Fixed	Non-fixed	Fixative effect (%)	*P value*
**Stigma area (mm2)**	10.24 ± 0.179	10.46 ± 0.178	-1.91	0.3855
**Ovary diameter (mm)**	2.49 ± 0.0324	2.57 ± 0.0324	-3.11	0.058

	**7 DAW9.5**			
	Fixed	Non-fixed	Fixative effect (%)	*P value*
**Stigma area (mm2)**	10.58 ± 0.242	10.78 ± 0.219	-1.85	0.5164
**Ovary diameter (mm)**	3.06 ± 0.0431	3.09 ± 0.0396	-0.97	0.479

	**13 DAW9.5**			
	Fixed	Non-fixed	Fixative effect (%)	*P value*
**Stigma area (mm2)**	8.34 ± 0.18	8.97 ± 0.18	-7.02	0.0132
**Ovary diameter (mm)**	3.47 ± 0.0328	3.49 ± 0.0418	-0.57	0.677

	**18 DAW9.5**			
	Fixed	Non-fixed	Fixative effect (%)	*P value*
**Stigma area (mm2)**	5.27 ± 0.178	6.35 ± 0.178	-17	<0.0001
**Ovary diameter (mm)**	3.71 ± 0.0328	3.63 ± 0.0339	2.2	0.0662

Note: P values shown for the different timepoints indicate the *P* values for the pairwise comparisons. Estimated marginal means ± standard errors are also given for each treatment (fixed or non-fixed).

Development • Supplementary information

Table S4. Expression data (raw transcript per million (TPM) values) for 42 senescence-associated genes in the wheat stigma of MS cultivar 24512 grown during the 2021 field season. In cases where a gene is below the 0.5 TPM detection criteria (see Methods) the value is ".". Information about each gene is also provided (i.e. Gene name, TF family and publication from which the candidate gene was selected).

Click here to download Table S4

Table S5. Output data obtained after running stigma CNN. Pixels were converted to mm2.

Click here to download Table S5

Table S6. Environmental data collected during 2021 field season.

Click here to download Table S6

Development • Supplementary information

Supplementary Materials and Methods

List of scripts and directories included in https://github.com/Uauy-Lab/ML-carpel_traits.

Scripts used to run stigma or ovary trained CNNs on raw .jpg data:

- measure-ovary.yml
- measure-stigma.yml
- requirements.txt
- analyse_ovary_diam_resumable.py
- analyse_stigma_area_resumable.py
- gather_working_dir.py
- gather_working_dir_area.py

Scripts can be found in "running_the_model" directory.

R script used to organise and visualise output data from stigma or ovary CNNs. An example is provided to analyse stigma area data:

- Stigma_area_script.R

Step-by-step guide: example for stigma area measurements

Download ML-carpel_traits repository by selecting "Download ZIP" under the "Code" button highlighted in green. Once downloaded you can decompress the folder.

For the following steps we used the command line in Git Bash. Git Bash can be downloaded from the following link: https://git-scm.com/downloads

Applying trained model to raw images to make stigma measurements

First, ensure you have a working version of Python 3, version 3.7 or later. You can do so by typing the following on the command line:

```
python --version
```

To install Python 3 on Windows 10 you can directly type `python3` on the command line and it will automatically be downloaded. Once the download is completed you can type `python --version` again to check Python 3 is now working.

Development • Supplementary information

Next, create a virtual environment:

```
cd ML-carpel_traits-main/running_the_model/

python3 -m venv venv

source venv/bin/activate
```

Then install the requirements:

```
pip install -r requirements.txt
```

If the installation of some requirements fails, you can manually do so as follows:

```
pip install dtoolai==0.2.0
```

Errors tend to be user-specific so we also recommend users to inspect the error messages that might appear during the installation as they normally suggest a way to solve the problem.

Create new directories for the raw images we want to analyse and output directories where the results will be saved:

```
mkdir -p input_folder_carpel #In this folder you should paste the
                              images

mkdir -p working_stigma #For ovary CNN, you need to create
                         working_ovary directory

mkdir -p results_stigma #For ovary CNN, you need to create
                         results_ovary directory
```

Once all the requirements have been successfully installed and additional directories created, we run the model as follows:

Stigma CNN

```
python scripts/analyse_stigma_area_resumable.py measure-stigma.yml

python scripts/gather_working_dir_area.py measure-stigma.yml
```

Ovary CNN

```
python scripts/analyse_ovary_diam_resumable.py measure- ovary.yml

python scripts/gather_working_dir.py measure-ovary.yml
```

Note: requirements need to be installed only the first time you run the model (independently of whether you run the stigma CNN or ovary CNN).

In “results_stigma” you should find a folder containing all the images annotated by the CNN and their annotations (expected path: running_the_model/results_stigma\output_folder_annotated_stigma/data). Additionally, you will find a “results.csv” file containing a filename column and a stigma_area_pixels column with the CNN measurements in pixels. Conversion to mm^2 will depend on the scale used for each image. We recommend acquiring all the images with the same magnification to ease the transformation of pixels to mm^2.

Verification of CNN annotated images

As previously mentioned (see discussion and Fig. 2E), we suggest the user to add a verification step to identify potential annotation errors before continuing with the downstream analyses. See Fig. S7 for examples. The user can decide whether to remove that image from the analysis or correct it by manually annotating the image using Fiji.

Organise and visualise output data

The R script Stigma_area_script.R can be used and modified to accommodate the research interests of the user. The aim of the script is to organise the output data, integrate additional field data (e.g., day temperatures) and generate ggplots illustrating the developmental dynamics of the carpel for the selected cultivars (Fig. S9). Rstudio is required to run the script. Note that the same script can be used for ovary diameter data by simply changing “stigma_area_mm” to, for example, “ovary_diam_mm”. In Supplementary Table 4 and 5 we provide data for two different MS cultivars as an example.

Development • Supplementary information
