# Supplementary material for: A scalable phenotyping approach for female floral organ development and senescence in the absence of pollination in wheat
Source: Development. 2022 Sep 15;149(18):dev200889. doi: 10.1242/dev.200889 (PMC9573784; doi:10.1242/dev.200889)
Supplement: Supplementary file 1 [file develop-149-200889_review_history.pdf]

A scalable phenotyping approach for female floral organ development and senescence in the absence of pollination in wheat

Marina Millan Blanquez, Matthew Hartley, Nicholas Bird, Yann Manes, Cristobal Uauy and Scott A. Boden

DOI: 10.1242/dev.200889

Editor: Yka Helariutta

Review timeline

Original submission: 26 April 2022
Editorial decision: 24 May 2022
First revision received: 8 July 2022
Accepted: 15 August 2022

Original submission

First decision letter

MS ID#: DEVELOP/2022/200889

MS TITLE: A scalable phenotyping approach for female floral organ development and senescence in the absence of pollination in wheat

AUTHORS: Marina Millan-Blanquez, Matthew Hartley, Nicholas Bird, Yann Manes, Cristobal Uauy, and Scott A Boden

I have now received all the referees' reports on the above manuscript, and have reached a decision. The referees' comments are appended below, or you can access them online: please go to BenchPress and click on the 'Manuscripts with Decisions' queue in the Author Area.

As you will see, the referees express considerable interest in your work, but have some significant criticisms and recommend a substantial revision of your manuscript before we can consider publication. If you are able to revise the manuscript along the lines suggested, which may involve further experiments, I will be happy receive a revised version of the manuscript. Your revised paper will be re-reviewed by one or more of the original referees, and acceptance of your manuscript will depend on your addressing satisfactorily the reviewers' major concerns. Please also note that Development will normally permit only one round of major revision. If it would be helpful, you are welcome to contact us to discuss your revision in greater detail. Please send us a point-by-point response indicating your plans for addressing the referee’s comments, and we will look over this and provide further guidance.

Please attend to all of the reviewers' comments and ensure that you clearly highlight all changes made in the revised manuscript. Please avoid using 'Tracked changes' in Word files as these are lost in PDF conversion. I should be grateful if you would also provide a point-by-point response detailing how you have dealt with the points raised by the reviewers in the 'Response to Reviewers' box. If you do not agree with any of their criticisms or suggestions please explain clearly why this is so.

© 2022. Published by The Company of Biologists under the terms of the Creative Commons Attribution License (https://creativecommons.org/licenses/by/4.0/).

Reviewer 1

Advance summary and potential significance to field

Millan-Blanquez et al present a new approach to study an overlooked process in cereal floral development – namely, what happens to the cereal carpel when not self-fertilised. The agronomic implications of post-anthesis carpel development and fertility potential are huge, not only for potential limits on yield but also on the window for hybrid production. The authors make a convincing case that this process is fundamentally interesting, genetically controlled and varies across the germplasm, and that building an ontogenic framework of this process will enable its functional and genetic dissection.

Their paper details how they developed and tested an automated image annotation method to quantify changes over time in ovary diameter and stigma area following anthesis of the unfertilised wheat carpel. They justify these traits as important based existing literature and precise preliminary manual annotation of 2D images of these phenotypes. Using a large image training set, they applied machine learning to construct convolutional neural network (CNN) to model these floral traits. They then tested their model on unseen images and validated the CNN output with manual annotation (measurements) of stigma size and ovary diameter. Lastly, they applied their CNNs to image data collected from three cultivars over two field seasons, revealing intriguing conservations of developmental stages as well as variation in stage transition timing between cultivars.

A real strength of this study is the breadth of input data and critical appraisals of their methods and computational outputs. For instance, the study used different conditions (glasshouse, field, and multiple year experiments) and multiple germplasm to develop and test their model. I especially appreciated their examination on the effects of fixation at all stages in post-anthesis carpel development. Taken together, the authors convince me that they conducted this research to an exceptionally high standard and that their model is robust across experimental conditions and germplasm.

I was also excited to read about their great success in applying machine learning to this problem. While I'm not best placed to evaluate the computational methods, the ground truth validation and DSC calculation suggest that their CNNs are robust. This serves as an excellent example of using validated computational algorithms to streamline phenotyping- enabling future studies to leap beyond current limitations to identity genetic basis for variation in traits. This approach will prove undoubtedly useful for researchers studying carpel development from all angles (fundamental to applied interest).

Comments for the author

The manuscript could be improved in a few places.

- I think some conclusions might be overstated – for instance, the authors suggest on lines 416-417 that the peak stigma area may represent maximum reproductive potential; although they reference earlier studies, could the authors expand on their justification for this idea? One could imagine a stigma which has maximum potential to capture pollen but not maximum potential for either pollen tube formation or successful fertilisation. Without further testing, I think the authors should tone down links about carpel image characteristics reflecting reproductive potential throughout their manuscript.
- I'd also like to understand more about the rationale of selecting the three MS cultivars for the developmental time course – do you think these cultivars capture the range or just a lucky dip? Do these cultivars contrast in other ways?
- I also found it very hard to follow which samples were used for which experiments – I think a table outlining the germplasm, experiment and output would help.
- Could the authors please describe the statistical tests referred to on line 211?
- While I appreciate that this technique is presented as the framework for future work and that this paper was submitted as a Techniques and Resources Article, I suspect that readers at Development will be looking for some demonstration of the tools' application to learn more about the critical events underlying each stage of carpel development in the absence of self-pollination either at the cellular, molecular or genetic level. Would the authors be able to provide some insight using existing samples and/or data? For instance expression of key genes involved in post-anthesis carpel development mentioned in the discussion (lines 526-528) or some anatomical data in any of their time course samples?

© 2022. Published by The Company of Biologists under the terms of the Creative Commons Attribution License (https://creativecommons.org/licenses/by/4.0/).

• I commend the authors on providing a comprehensive and freely available access to data, training sets and scripts. However, it would be more useful for the wider community to include a step by step protocol on how to use this tool and the skills and packages needed (i.e. R studio, familiarity with R, notes about which parameters need to be revised in the script etc.). Given the need for programming experience, I'm concerned about this tool's uptake in the wider community without more user instructions and guides.

Reviewer 2

Advance summary and potential significance to field

The article provides a new tool, based on AI, to speed up phenotyping of carpel characters in wheat likely linked to the period where the carpel is receptive to fertilization. It is hard for me to comment on the CNN development and the algorithms used, because I am absolutely no expert in these. However, I can comment about the technical and methodological aspects of phenotyping. In my opinion, the work has been done with high standards of rigor and covering very well the relevant aspects (enough number of genotypes, environmental variables, the use of fixatives or fresh tissue...). All has been done carefully and provide a solid ground for the claims of the usefulness of the technique and its potential in advancing breeding programs.

Comments for the author

I am limited by my poor ability to assess the AI approach, so I cannot suggest any improvement in that sense. About the mor "biological" part, the only thing that I do miss is a stronger correlation of carpel morphological traits (throughout the phases described) and effective fertility. An estimation of fertilization potential could have been done in the studied genotypes to assess whether the morphology of the carpel is tightly correlated with fertility or this also varies in the different genotypes.If the correlation is not almost perfect, the usefulness of the approach could be quite reduced.

Apart from this, I cannot find weak points that should be improved

---

First revision

Author response to reviewers' comments

REVIEWER 1

• *I think some conclusions might be overstated – for instance, the authors suggest on lines 416-417 that the peak stigma area may represent maximum reproductive potential; although they reference earlier studies, could the authors expand on their justification for this idea? One could imagine a stigma which has maximum potential to capture pollen but not maximum potential for either pollen tube formation or successful fertilisation. Without further testing, I think the authors should tone down links about carpel image characteristics reflecting reproductive potential throughout their manuscript.*

We thank the reviewer for their comment and agree that additional experiments, which are beyond the scope of this current study, are needed to test the hypothesis that there is a connection between the peak phase of stigma/ovary growth and maximum receptive potential. Our intention was always to present this as a hypothesis that will lead to future research rather than a definitive conclusion, and we can now test this prediction using the platform developed here. We have now expanded on the basis of our hypothesis regarding a possible link between the peak phase and maximum receptive potential of the carpel and provided future directions that we are currently following to test this idea under field conditions (lines 440 to 450). Together with the section of the discussion entitled “First steps towards an integrated developmental scale of the unpollinated

© 2022. Published by The Company of Biologists under the terms of the Creative Commons Attribution License (https://creativecommons.org/licenses/by/4.0/).

wheat flower", we believe the text now provides the right degree of caution about the link between morphology and receptivity and reiterates that future studies are needed to test this hypothesis.

- *I'd also like to understand more about the rationale of selecting the three MS cultivars for the developmental time course – do you think these cultivars capture the range or just a lucky dip? Do these cultivars contrast in other ways?*

We thank the reviewer for this very pertinent question. We based the selection of these three cultivars on the amount of variation in stigma area and ovary diameter observed within the original pool of 31 MS cultivars phenotyped during the 2020 field season (Figure R1 below). We did not include this information in the manuscript for simplicity as our main objective was to showcase the potential of the approach to capture phenotypic variation in these two floral traits, and that indeed, there is a substantial degree of phenotypic and genotypic variation for these traits in wheat. We have included new text in the Materials and Methods section that explains why we selected those three cultivars (lines 111-113).

Note: *We have removed unpublished data that had been provided for the referees in confidence.*

Figure R1: Developmental dynamics of stigma area in the absence of pollination for 31 MS wheat cultivars in the 2020 field season. Experimental design as described in Materials and Methods for field season 2020.

- *I also found it very hard to follow which samples were used for which experiments – I think a table outlining the germplasm, experiment and output would help.*

We thank the reviewer for their excellent suggestion, and we have now included a new Supplementary Table (Supplementary Table 1) with information outlining the output and germplasm used for each experiment, referred to in lines 111 and 112.

- *Could the authors please describe the statistical tests referred to on line 211?*

We thank the reviewer for their question. We realised that the sentence (line 211) was not completely accurate as the scripts we provide do not include any statistical analysis. We have now rearranged that paragraph to clarify the statistical tests used here, which relates to the information provided on lines 190-192.

- *While I appreciate that this technique is presented as the framework for future work and that this paper was submitted as a Techniques and Resources Article, I suspect that readers at Development will be looking for some demonstration of the tools' application to learn more about the critical events underlying each stage of carpel development in the absence of self-pollination, either at the cellular, molecular or genetic level. Would the authors be able to provide some insight using existing samples and/or data? For instance, expression of key genes involved in post-anthesis carpel development mentioned in the discussion (lines 526-528) or some anatomical data in any of their time course samples?*

We thank the reviewer for their suggestion. To address this comment, we now include data belonging to a separate unpublished study where we conducted an RNA-seq transcriptome experiment of developing stigmas during the field season of 2021, using MS cultivar 24512. These data include a subset of senescence associated genes like *KIR1* and *ORE1* (mentioned in the discussion), alongside other genes linked to senescence in wheat, to illustrate the proposed phases of late carpel development are linked to expected changes in gene expression. This new information is presented in the Results as a heatmap of gene expression (Fig. 5E) and is explained in lines 493 to 510. We have included text in the introduction and discussion to contextualise these results in reference to the peak and deterioration phases of stigma growth (i.e., lines 65-67 and 575-576), and we have outlined the relevant experimental procedure in the materials and methods ("Stigma transcriptome analysis by RNA-seq"). We wish to point out that we have not included all of the raw data for the stigma transcriptome of MS24512 in this submission, as the data are part of

© 2022. Published by The Company of Biologists under the terms of the Creative Commons Attribution License (https://creativecommons.org/licenses/by/4.0/).

another broader study that we wish to publish independently. Nonetheless, we have provided the individual expression values for the set of genes referred to in the manuscript, which is provided in Supplementary Table 4.

- *I commend the authors on providing a comprehensive and freely available access to data, training sets and scripts. However, it would be more useful for the wider community to include a step by step protocol on how to use this tool and the skills and packages needed (i.e. R studio, familiarity with R, notes about which parameters need to be revised in the script etc.). Given the need for programming experience, I'm concerned about this tool's uptake in the wider community without more user instructions and guides.*

We really appreciate the reviewer's suggestion, and we have now incorporated a section under Supplementary materials and methods where we provide a step-by-step guide on how to install and run the described models. We have also included an exemplar dataset (Table S5 and S6) imitating what the user will obtain after running the model and the output from running the R script on the data (Fig. S9). In the manuscript, we refer to this new section in lines 217-218.

REVIEWER 2

About the more "biological" part, the only thing that I do miss is a stronger correlation of carpel morphological traits (throughout the phases described) and effective fertility. An estimation of fertilization potential could have been done in the studied genotypes to assess whether the morphology of the carpel is tightly correlated with fertility or this also varies in the different genotypes. If the correlation is not almost perfect, the usefulness of the approach could be quite reduced.

We totally understand the value that including fertility data (e.g., seed set) would add to the proposed phases. However, this type of experiment is not only complex to perform under field conditions, but is also difficult to interpret if free pollination, for instance, is used. Hybrid seed production in the field is influenced by a wide range of factors that extend beyond stigma receptivity, or even female receptivity, if we also consider the ovary. For instance, wind speed at the time of pollination, pollen availability and viability, anther extrusion, plant height, and or spike architecture, just to mention a few. Although we started to perform fertility experiments in the field, we are still optimising the experimental set-up to account for variables such as weather conditions. It is for this reason that we have not included fertility data in this study as we expect to be able to make this connection in a follow-up study once we have reliable multi-year fertility data. As mentioned, we do understand the importance and value of creating this link between morphology and fertility, so we have included more text outlining future directions to test this (currently) hypothetical link (lines 440 to 450). As reviewer 1 commented similarly about this aspect of the study, we have now provided gene expression data to show that the proposed phases of late carpel development are linked to changes in the transcription of genes that influence stigma fertility and senescence (lines 493-510).

Second decision letter

MS ID#: DEVELOP/2022/200889

MS TITLE: A scalable phenotyping approach for female floral organ development and senescence in the absence of pollination in wheat

AUTHORS: Marina Millan-Blanquez, Matthew Hartley, Nicholas Bird, Yann Manes, Cristobal Uauy, and Scott A Boden
ARTICLE TYPE: Techniques and Resources Article

I am happy to tell you that your manuscript has been accepted for publication in Development, pending our standard ethics checks.

© 2022. Published by The Company of Biologists under the terms of the Creative Commons Attribution License (https://creativecommons.org/licenses/by/4.0/).

Reviewer 1

Advance summary and potential significance to field

Millan-Blanquez et al revision addresses many of my concerns about the manuscript's first draft and I thank the authors for their efforts revising their paper. The revised manuscript contains additional cautions about correlating maximum reproductive potential and stigma development which addresses my concerns (nb at lines 252 to 258 in the version I have to review, rather then 440 to 450). The rearrangement and clarification about the scripts and statistics, as well as the added table and text work well to explain each experiment and corresponding germplasm. The revised manuscript now includes gene expression along growth, peak and senescence phases from a cultivar used in the multiyear field trial. I was intrigued to see the upregulation of the senescence-related transcription factors within (although towards the end) of the peak phase, suggesting anticipatory changes in gene expression. The new work also highlights an interesting link between leaf and stigma senescence. Altogether these new analyses address my original comment and work well to demonstrate and validate their sampling. I also think the new step-by-step, along with the exemplar dataset and expected outcome, should help other scientists use their approach and address Development's aim that tools be described for broad uptake and applicability.

Taken together, the revised manuscript clarifies and expands on this understudied area, and provides technical and computational foundations to understand this crucial window of stigma reproductive development.

Comments for the author

I have just a few minor comments.

- Including justification in the methods addresses my concerns about cultivar selection but I would encourage the authors to include these details in the results section as well.
- I suggest that the step-by-step guide be mentioned in the main text rather than the Methods section.
- In Figure 5, I suggest labelling the cultivar on the bar sliders below the line graphs in Figure 5C. Also, the font used to label columns in heat map seems elongated to me.

Reviewer 2

Advance summary and potential significance to field

As I said in my assessment of the first version, I find these tools useful for breeding and in general for the field and I support publication based on novelty of approaches and rigor of the protocols

Comments for the author

I do think that the authors have made Ann effort to address minor issues that were brought about by reviewers and the improvements introduced to the manuscript are more than enough to grant publication. I do not find additional issues that need to be considered

© 2022. Published by The Company of Biologists under the terms of the Creative Commons Attribution License (https://creativecommons.org/licenses/by/4.0/).
